# Dynamics of long-term genomic selection

**DOI:** 10.1186/1297-9686-42-35

**Published:** 2010-08-16

**Authors:** Jean-Luc Jannink

**Affiliations:** 1 USDA-ARS, RW Holley Center for Agriculture and Health, Ithaca, NY 14853, USA; 2 Cornell University, Department of Plant Breeding and Genetics, Ithaca, NY 14853, USA

## Abstract

**Background:**

Simulation and empirical studies of genomic selection (GS) show accuracies sufficient to generate rapid gains in early selection cycles. Beyond those cycles, allele frequency changes, recombination, and inbreeding make analytical prediction of gain impossible. The impacts of GS on long-term gain should be studied prior to its implementation.

**Methods:**

A simulation case-study of this issue was done for barley, an inbred crop. On the basis of marker data on 192 breeding lines from an elite six-row spring barley program, stochastic simulation was used to explore the effects of large or small initial training populations with heritabilities of 0.2 or 0.5, applying GS before or after phenotyping, and applying additional weight on low-frequency favorable marker alleles. Genomic predictions were from ridge regression or a Bayesian analysis.

**Results:**

Assuming that applying GS prior to phenotyping shortened breeding cycle time by 50%, this practice strongly increased early selection gains but also caused the loss of many favorable QTL alleles, leading to loss of genetic variance, loss of GS accuracy, and a low selection plateau. Placing additional weight on low-frequency favorable marker alleles, however, allowed GS to increase their frequency earlier on, causing an initial increase in genetic variance. This dynamic led to higher long-term gain while mitigating losses in short-term gain. Weighted GS also increased the maintenance of marker polymorphism, ensuring that QTL-marker linkage disequilibrium was higher than in unweighted GS.

**Conclusions:**

Losing favorable alleles that are in weak linkage disequilibrium with markers is perhaps inevitable when using GS. Placing additional weight on low-frequency favorable alleles, however, may reduce the rate of loss of such alleles to below that of phenotypic selection. Applying such weights at the beginning of GS implementation is important.

## Background

Simulation studies and some empirical studies of "genomic selection" (GS) [1] or "genome-wide selection" [2] show that prediction accuracies from GS are high enough to enable rapid gains from selection [3-6]. These studies focus, however, on what would be the first one or two cycles of selection. Thus, while we may have confidence that GS can accelerate short-term gain, no such confidence is justified for long-term gain. Ideally, experimental tests of long-term gain should be performed empirically in model systems but the necessary replicated tests would be expensive and, even in rapid-cycling organisms, would not be completed in a near future. Stochastic simulation remains perhaps the only viable option to test hypotheses concerning the impact of selection methods on long-term gain [7].

Beyond the first cycles of selection, mechanisms the effects of which are difficult to predict analytically begin to operate. Among others, marker and QTL alleles will recombine, and their frequencies will shift, changing linkage disequilibrium (LD) between them and therefore the predictive ability of the markers. Inbreeding and loss of polymorphism will also occur. In a simulation looking at several generations, Muir [8] has shown that the accuracy of genomic prediction declines much more rapidly if used for selection than if followed by random mating. This result and the putative mechanisms outlined suggest that a careful look at long-term selection using GS is needed to identify mechanisms having an important impact on its performance and to give research directions to improve GS. There is also a practical need since both crop and animal breeding programs are now initiating GS. Therefore, insight into the long-term consequences of GS deployment would be beneficial.

Considering the constraints of breeding cycles over several generations also brings into focus practical aspects of GS that have a bearing on its potential for success. In particular, Heffner et al. [9] have proposed that GS separates the breeding process into two cycles: the selection cycle and a model training cycle. They have proposed that these two cycles operate synchronously, although this is not necessarily the case. The model training cycle is much more constrained than the selection cycle because it requires adequate phenotyping. Thus, regardless of the species, it appears likely that the frequency of model updating will be lower than that of selection cycles. This limitation raises the questions of how accurate GS can be in selection cycles when it has not been updated, and to what extent long-term selection will be adversely affected.

Another constraint for GS is the necessity of assembling the initial training population (TP) for the model. In simulations using population-wide LD, rather large TP have been used i.e. 500 to 2000 individuals [1,10,11]. In GS on bi-parental cross populations, much smaller populations have been effective [4,12], though these populations have never been proposed for long-term selection. Therefore the question arises of the effectiveness of GS if cost prohibits assembling a large TP and GS is initiated on the basis of a small TP.

Finally, different GS prediction models have been proposed the impacts of which may differ on the short and long terms. In simulations of generations immediately after the TP, models that assume all marker effects are distributed with equal variance (i.e. ridge regression), have been found to be as or more accurate than models that assume some markers do not explain any variance (e.g. BayesB) [1]. However, the accuracy of the former decays more rapidly over generations than that of the latter [10]. How this dynamic may affect the performance of these models over long-term selection is unknown.

To explore the questions of long-term success of GS, impact of initial training population size, timing of additions of new phenotypes to the training population, and on GS analysis method, long-term selection for a quantitative trait using GS was simulated. Gains from GS were compared to those of phenotypic selection (PS). Genomic selection was performed on lines with or without phenotypes, and assuming cases where phenotyping (and therefore model updating) could occur every or only every other selection cycle. Gains using an initial TP of 1000 vs. 200 individuals were compared. Ridge regression was contrasted to a Bayesian model for GS prediction, and marker effects weighted by a function of favorable allele frequencies were compared to unweighted effects. Finally, to understand the mechanisms leading to GS success or failure, population variables were analyzed including the maintained genetic variance, realized accuracies, LD and distance between QTL and markers remaining polymorphic, inbreeding over generations and the fixation of QTL and marker alleles.

## Methods

### Barley data set

To perform selection simulations on marker data that incorporate the real short- and long-range LD structure existing in a barley breeding program, empirical genotypes from 192 inbred lines from the University of Minnesota six-row spring barley breeding program (genotyped in the first two years of the Barley Coordinated Agricultural Project) were used. These marker data may be obtained at http://www.hordeumtoolbox.org. Missing marker data were imputed using methods described by Jannink et al. [13] on the basis of the SNP genetic map given by Close et al. [14]. Markers were considered redundant if they had the same map position and identical alleles across all lines. Only one of a set of redundant markers was retained. This procedure left 983 polymorphic markers among the Minnesota lines. Some sets of markers mapped to the same position, most likely because of insufficient resolution of bi-parental maps rather than because of actual identical positions [14]. Markers in such sets were distributed at 0.1 cM intervals and in arbitrary order. The resulting map spanned 1,092 cM.

### Genetic model

An additive genetic model was imposed on these marker data by randomly picking 100 markers to become causal QTL. These markers were removed from the dataset for GS analyses. The genetic variance generated by each QTL was made equal by scaling the QTL substitution effect to the inverse of the standard deviation of the QTL allelic state (+1 for one and -1 for the other allele). Thus, QTL with low minor allele frequencies (MAF) had larger substitution effects than QTL with high MAF. This constraint of equal variance across QTL was chosen to maximize the effective QTL number [15] while minimizing the number of markers that had be dropped from the analysis. Empirical genomic selection results suggest that many traits are more polygenic than what was simulated previously [3]. One QTL allele was arbitrarily chosen to have a positive, and the other a negative effect. The genotypic value of an individual was calculated by summing effects of the QTL alleles it carried. The phenotype of an individual was determined by adding its genotypic value to a normally distributed error, with variance calculated as follows. The genotypic variance of the base population was calculated and an error variance determined so that the initial trait heritability was either 0.2 or 0.5. Error variance was held constant through a simulation irrespective of changes in the genetic variance, such that heritability changed over the course of generations of selection.

### Stochastic simulations

For all simulated breeding methods, each cycle of breeding consisted of three steps: (1) crossing of selected parents and inbred progeny generation, (2) phenotyping and (3) data analysis and selection criterion estimation. For all methods, step 1 was the same: out of 200 candidates, the 20 with the highest selection criterion were randomly mated to produce 200 F_1 _progeny. Inbred selection candidates were generated as doubled haploids (DH) from the F_1 _generation. Random mating is not a realistic assumption for breeding but it provides a simple baseline model to interpret results. While inbreeding is not needed for genomic selection, it is needed in crop breeding for phenotypic evaluation. For simplicity, inbreeding was performed prior to selection for all schemes. Each DH was formed from a haploid gamete simulated using the Mendelian laws of segregation, with recombination occurring according to the known map positions of the barley markers [14], assuming no crossover interference. For all methods, the base population was formed by randomly mating the 192 founders to generate 200 DH candidates that were phenotyped, as described above. For GS with a "small" TP, this base population served as the TP. For GS with a "large" TP, an additional 800 individuals were generated and phenotyped in the same way. While these individuals provided information to the GS model, they were not selection candidates. Thus, the training population size factor was not confounded with a change in selection intensity.

**Figure 1:**
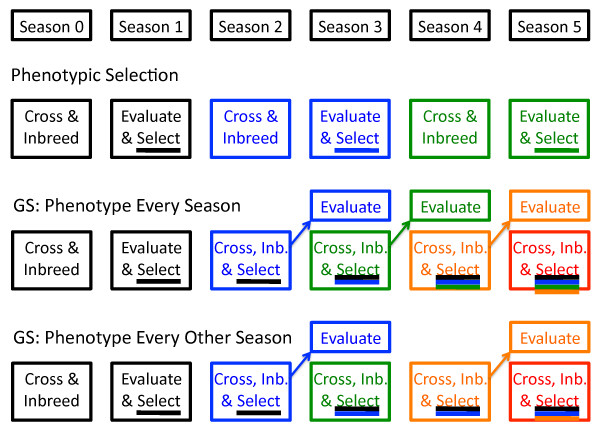
**Phenotypic and genomic selection breeding schemes**. Under phenotypic selection, one season is used for crossing and inbreeding and the next for evaluation and selection; under GS, selection can be performed prior to evaluation so that selection occurs every season rather than every other season; for "every season phenotyping," the evaluation is assumed to represent the target environment in any season; for "every other season phenotyping," only odd-numbered seasons represent the target environment and even-numbered seasons are greenhouse or off-season nurseries; for all methods, the black cycle (C0) is phenotyped; for GS, this cycle contributes to the training population (TP), as indicated by the colored line under the word "Select" in Season 1; in Season 2, candidates of the blue cycle (C1) are produced, and selection is possible under GS, but using the same TP as for Season 1 (insufficient time for new phenotyping has elapsed); in Season 3, candidates of the green cycle (C2) are produced, evaluation of C1 candidates occurs and can contribute to the TP used to select C2 candidates; similar events occur in Season 4 except that for every other season phenotyping, evaluations are not performed because they would not be representative of the target environment

Phenotypic selection and three GS breeding schemes were simulated. Time was somewhat arbitrarily broken up into "seasons" with PS requiring two seasons, one for crossing and inbred candidate generation, and one for phenotypic evaluation and selection (Figure 1). In the first GS scheme, all candidates were phenotyped and genotyped so that the model had both sources of information available. This "genomic and phenotypic selection" (GPS) scheme followed the time schedule of PS (Figure 1). In the two GS schemes, selection occurred solely on marker data immediately after, and in the same season as, inbreeding (Figure 1). In the "phenotype every season" GS scheme, candidates were then phenotyped in the following season to supplement the TP. In the "phenotype every other season" GS scheme, it was assumed that only odd-numbered seasons allowed phenotyping in the target environment (Figure 1). Therefore, only selection candidates from even-numbered seasons had to be phenotyped to supplement the TP. To ensure that all GS methods involved the same amount of phenotyping, only 50% of "phenotype every season" candidates were phenotyped (since phenotyping occurred in twice as many seasons). The 50% chosen for phenotyping were those that had the highest selection criterion of their cohort. Thus the parents selected to perpetuate the breeding cycle were always phenotyped.

### Genomic selection prediction models

Two prediction models were used, ridge regression i.e. RR [1,10] and "BayesC*π*" (RL Fernando, personal communication, June 2009). Both RR and BayesC*π *use the linear model

where *y*_*i *_is the phenotype of individual *i*, *x*_*ij *_is the allelic state at marker *j *in individual *i*, *β*_*j *_is the effect associated with marker *j*, *δ*_*j *_is a 1 or 0 indicator variable for the inclusion or exclusion of marker *j *in the estimation of breeding values, and *e*_*i *_is a residual. In RR, *δ*_*j *_= 1 and  for all markers. The marker variance, , is estimated by maximum likelihood. BayesC*π *implements two changes relative to BayesB developed by [1]. As in BayesB, in BayesC*π*, *δ*_*j *_= 0 with probability *π*, but *π *itself is estimated assuming a uniform prior distribution between 0 and 1. In addition, BayesC*π *assumes that the prior variance for the effects of all markers for which *δ*_*j *_= 1 is equal. That is, the effect *β*_*j *_is zero when *δ*_*j *_= 0 or  when *δ*_*j *_= 1. In turn, the method estimates  jointly over all non-zero markers [16]. Grouping markers in this way gives the data added weight over the prior in estimating [17]. Details of the estimation of  are in Kizilkaya et al. [16]. The model provides an estimate of marker effects as

where *T *is the number of Markov chain iterations and *β*_*jt *_and *δ*_*jt *_are the values for those parameters in iteration *t*. Here, 1500 iterations were run, with the first 500 discarded as burn-in.

Using these models, genomic prediction in a given breeding cycle was performed by analyzing the marker states of all individuals with phenotypes to estimate marker effects. These effects were then applied to the genotypes of selection candidates to predict their breeding values:

Finally, a weighted GS model was used, following Goddard [18] and clarified in Hayes et al. [6] so that markers for which the favorable allele had a low frequency should be weighted more heavily to avoid losing such alleles. For weighted GS, the estimation procedure was as described above. Then, for each marker *j*, the frequency of the favorable allele among selection candidates, *p*_*j*_, was calculated. The selection criterion was

Using  as a weight for locus *j *is a simplification with the following justification. Using Goddard's optimization [18], assuming sufficient long-term selection to fix all favorable alleles, the selection criterion should be:

This criterion includes only the sign (positive or negative) of the locus effect, because it is assumed that the favorable allele should be fixed regardless of the magnitude of its effect.

 is closely proportional to  over a range of allele frequencies. In addition, an estimate of the allelic effect was included in the criterion to reduce the importance of small-effect loci for which it could not be determined with any certainty which allele was favorable.

In summary, 48 different GS schemes were tested: a factorial of two heritabilities (0.2 or 0.5), two initial TP sizes (200 or 1000), three breeding schemes (with phenotyping prior to selection, phenotyping after selection every season, or every other season), two prediction models (RR or BayesC*π*), and unweighted or weighted allele effects. In addition, simple phenotypic mass selection was simulated at heritabilities of 0.2 or 0.5. All settings were replicated 100 times. Replications differed in the base population of 200 individuals generated by randomly mating the 192 founder lines and in the 100 markers chosen to be QTL and removed from the marker dataset. Twenty seasons were simulated. For phenotypic selection (PS) and genomic and phenotypic selection (GPS) schemes, ten breeding cycles could be accomplished, while for the two GS cycles, 19 could be accomplished (one in the first two seasons and then one per season for the remaining 18 seasons). All simulations were performed in R, version 2.10 [19].

### Analysis of simulation results

For each simulation, gains from selection were standardized by dividing by the maximal genotypic value possible for the genetic model. Therefore for all replications, genotypic values are expressed on a -1 to +1 scale. Besides the mean genotypic values of selected populations, other tracked variables were additive genetic standard deviations, rates of inbreeding calculated on the basis of pedigree (ΔF_P_; in a pedigree with DHs, the standard tabular method for calculating coancestries can be used, save that all diagonal elements are set to one), Bulmer effects (calculated as the ratio of the additive genetic standard deviation to the expected additive genetic standard deviation under linkage equilibrium between QTL), and the realized accuracies in each generation of selection, which was calculated as

where G(t) is the mean genotypic value in generation *t*, *σ*_*A*_(*t*) is the additive genetic standard deviation in generation *t*, and 1.755 is the mean of the upper 10% tail of a standard normal distribution [20]. Several variables were tracked to examine mechanisms causing the observed responses: the number of favorable QTL alleles lost or fixed, the mean across polymorphic QTL of each QTL's LD with that marker with which it was in highest LD (LD was calculated here as the correlation between QTL and marker), the mean across polymorphic QTL of each QTL's recombination frequency with the closest polymorphic marker, and the ratio between the rate of inbreeding calculated on the basis of markers (ΔF_M_) and ΔF_P_. The rate of inbreeding on the basis of markers was calculated as the proportion of markers polymorphic in generation *t *- 1 that were fixed in generation *t*. Analysis of variance was performed on cumulative gain from selection after four seasons (two PS or GPS and three GS cycles, Figure 1) and after twenty seasons (ten PS or GPS and 19 GS cycles). Because 100 replications of each setting were performed, the power to identify "significant" interactions among simulation factors was very high. Therefore only interactions for which the mean square was at least one tenth that of the mean square for replications are discussed.

## Results

Under the simulated conditions, differences in both initial and final gain from GS using RR versus BayesC*π *were extremely small, though BayesC*π *tended to generate higher initial gains and lower final gains than RR (data not shown). Under GS, the difference between phenotyping half of the selection candidates every season versus all candidates every other season were minimal. Because these two factors (GS prediction method and every vs. every other season phenotyping) had effects that were small relative to between-replication variation, the discussion hereafter will focus on simulations using RR and phenotyping all candidates every other season.

**Figure 2:**
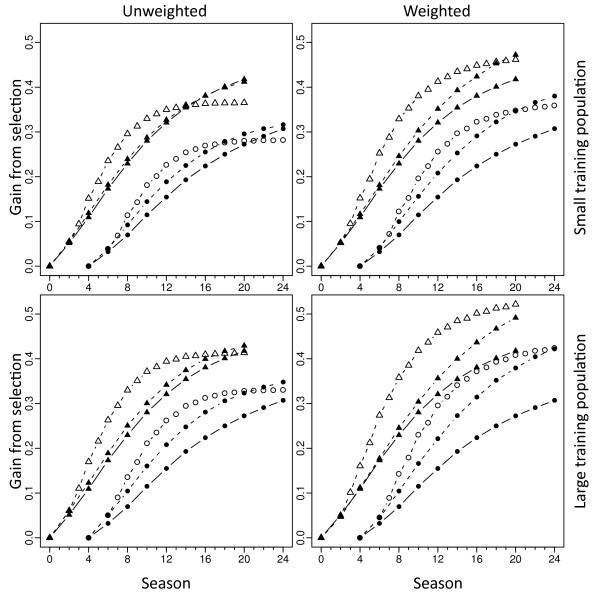
**Gain from phenotypic and genomic selection**. Phenotypic selection (PS, closed symbols, continuous lines), genomic selection with phenotyping prior to selection (GPS, closed symbols, dashed lines), and genomic selection (GS, open symbols, dashed lines), using ridge regression to estimate genomic breeding values. Weighted and unweighted methods were used for GS and GPS, on the right- and left-hand graphs, respectively; small and large training populations were of 200 and 1000 individuals, on the upper and lower graphs, respectively; triangles: h^2 ^= 0.5; Circles: h^2 ^= 0.2; to avoid cluttered graphs, simulations with h^2 ^= 0.2 were offset to the right by four seasons; note that PS curves are identical across the four graphs; maximum standard errors observed were less than half the height of plot symbols so no error bars are given

Looking first at unweighted GS (UGS; left-hand graphs of Figure 2), several points are apparent. First, performing selection every season (i.e., by selecting prior to phenotypic evaluation) always increased initial gain relative to waiting for evaluation results (i.e., using PS or GPS with selection only every other season). Second, phenotyping prior to selection increased long-term gain: after 20 seasons, rate of gain from PS and GPS was higher than that from GS. In fact, regardless of a high or low heritability, small or large TP, after about 12 cycles, GS reached a plateau beyond which gains were minimal (Figure 2). At a high heritability, genotypic information used by GPS hardly improved gain over PS. Besides, greater initial gains were obtained under a high than a low heritability for GS, leading to a significant GS vs. GPS by heritability interaction. Finally, having a large TP increased gain both for GS and GPS, but more so for the former, again leading to a significant interaction. Weighted GS (WGS; right-hand graphs of Figure 2) increased final gain from selection. Less apparent but no less important, weighting hardly changed initial gain, showing little tradeoff between long- and short-term gains. Weighting was more important in the absence of phenotyping prior to selection: it improved GS gains more than GPS gains. Weighting also produced greater gains with the large than with the small TP. Finally, weighting increased gains more at a high heritability than at a low one.

From these results, two observations bear further scrutiny. First, why did gains from selection reach a plateau so early under UGS, regardless of TP size and heritability? Loss of genetic variance and/or loss of LD between markers and QTL could be responsible. Second, what mechanisms contributed most to the performance of WGS? Here, QTL and/or marker polymorphism could be important.

**Figure 3:**
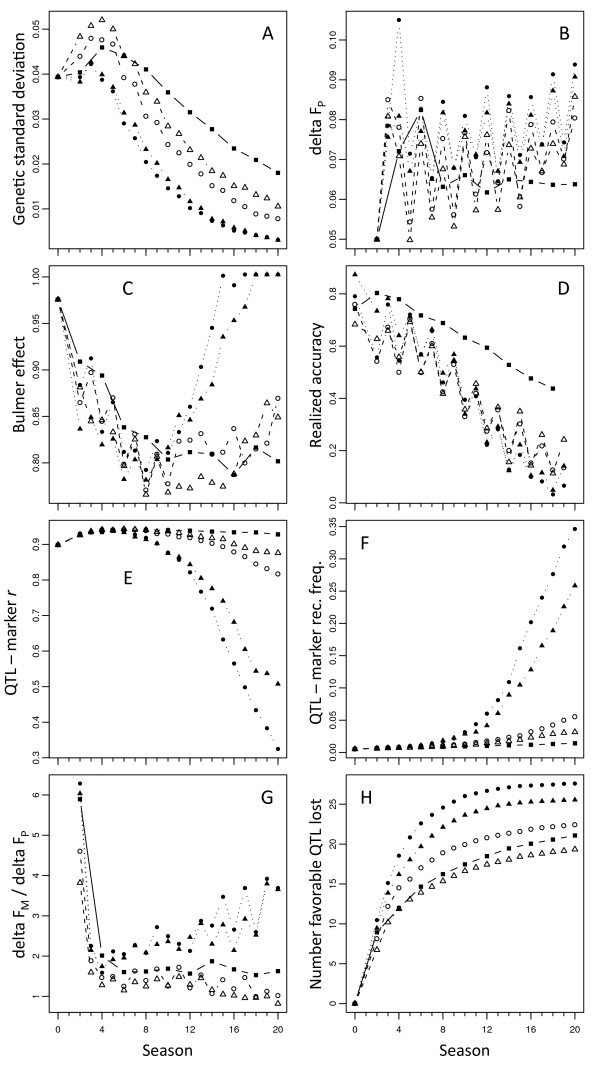
**Variables affecting long-term response to genomic selection**. Simulations at heritability of 0.5 using ridge regression to estimate breeding values and model updating every other season. Squares and continuous lines: phenotypic selection; circles vs triangles: small vs. large training population; closed vs open: unweighted vs. weighted GS; seasons correspond to the scheme given in Figure 1; A. genetic standard deviation among selection candidates in each cycle; B. rate of inbreeding calculated on the basis of pedigree, ΔF_P_; C. Bulmer effect, given by the ratio between observed genotypic standard deviation and that expected under linkage equilibrium; D. realized accuracy of selection; E. mean absolute correlation between QTL and markers in highest LD with them; F. mean recombination frequency between QTL and markers closest to them; G. ratio between rate of inbreeding calculated on the basis of markers (ΔF_M_) to that on the basis of pedigree; H. number of favorable QTL alleles lost from the selection population

The most immediate cause of the plateau reached by UGS is the loss of genetic variance in UGS populations (Figure 3A). This loss was more pronounced for the small than for the large initial TP but in either case was much stronger for UGS than for WGS. Increased weight on rare favorable marker alleles led to more rapid gains in the frequency of rare favorable QTL alleles with which only those markers could be in high LD. That impact on the QTL then strongly increased genetic standard deviation in the first cycles (Figure 3A). The proportional increase in gain explains why little short-term gain from selection was lost under WGS (Figure 2). The loss of variance came primarily from inbreeding (Figure 3B). The per cycle rate of inbreeding from UGS was generally higher than that of PS, while that of WGS was similar (Figure 3B). More importantly, GS went through twice as many cycles as PS, so that the per season rate of inbreeding was much higher. Two other observations on inbreeding rates bear note. First, in seasons when the prediction model was updated (odd-numbered seasons, Figure 1), ΔF_P _is consistently lower than in seasons when the model is not updated, leading to the zigzag pattern in ΔF_P _over selection cycles (Figure 3B). This zigzag pattern is counter-cyclical to that observed in the realized accuracies (Figure 3D) in the sense that when realized accuracy is up, ΔF_P _is down, and vice-versa. Second, for both WGS and UGS, there is a trend upward in ΔF_P _over time. This trend also corresponds to a general downward trend in realized accuracies (Figure 3D). Estimates of the Bulmer effect were noisier (Figure 3C). A zigzag pattern was also present: the Bulmer effect was stronger in the generation after model updating, that is, after realized accuracy was the strongest. For both UGS and WGS, the Bulmer effect diminished (leading to ratios closer to 1) when genetic variance diminished. Despite lower accuracies for GS than PS (Figure 3D), the Bulmer effect appeared stronger for the former than the latter.

Another possible cause for decrease in the accuracy of GS predictions is decay of marker - QTL associations. This decay began for UGS after about the eighth season and then strongly accelerated after that (Figure 3E). In contrast, for WGS, the decay in QTL - marker LD did not start until several seasons later and remained mild. Decay might arise because markers close to QTL become fixed such that the distance between the nearest polymorphic marker to a polymorphic QTL increases, and recombination more rapidly reduces accuracy. That mechanism indeed occurred (Figure 3F), again, much more strongly for UGS than WGS. Figure 3F also shows that marker fixation per season is more rapid under GS (both weighted and unweighted) than under PS: because GS selects on markers, it is more likely to cause markers to go to fixation than PS. Mechanistically, however, it is instructive to look at the rate of marker fixation relative to the rate of inbreeding. Figure 3G contrasts the proportion of polymorphic markers becoming fixed in each generation to the rate of inbreeding based on pedigree. Phenotypic selection provides an expectation for how much marker fixation to expect for a given increase in coancestry. Marker fixation occurs more rapidly than increase in identity by descent because a marker can become "fixed" when all its alleles are identical in state, which may occur before they are all identical by descent. Thus the equilibrium of the ratio of the rate of marker fixation to the rate of inbreeding is greater than one. In the case simulated here, that equilibrium for PS was about 1.7. For UGS, marker fixation clearly occurred more rapidly than might be expected on the basis of increasing coancestry (Figure 3G). In contrast, for WGS, marker fixation appeared to occur more slowly than expected on the basis of coancestry, at least in the later seasons. Thus, WGS might keep markers "in play" by selecting more strongly on low frequency alleles if they are associated with favorable QTL alleles. The bottom line of selection is to avoid the loss of favorable alleles, so that they may ultimately become fixed. A large loss in the number of favorable QTL alleles occurred in the first generation (Figure 3H), but that loss was smaller for WGS than for either UGS or PS. In the two subsequent seasons, per-season loss of favorable alleles was higher for both UGS and WGS than for PS. Thereafter, that higher rate of loss continued for UGS but slowed for WGS such that the rate of loss was lower for WGS than PS.

## Discussion

Before discussing results in detail, we should consider aspects of the simulation that lack realism and the impact those aspects might have on results. One strength of the marker data used here is that they reproduce levels of LD and a structure that occur within a real breeding program. However, true QTL were unobserved and simulating them using marker data is likely unrealistic for several reasons. First, this approach forces the QTL to be bi-allelic. Evidence is lacking in inbred crops with a small effective population size (N_e_) but in maize, an outcrosser with a large global N_e_, a recent study has shown that multi-allelic QTL are the norm [21]. It seems probable that multi-allelic QTL would be in lower LD than bi-allelic QTL with bi-allelic markers. Lower LD would in turn reduce the performance of GS relative to PS, though it is unclear how it would affect the relative performances of different GS schemes. Second, the approach means that the QTL have the same allele frequency spectrum as the markers, and the same distribution over the genome. Again, these limitations mean that the simulated QTL are probably in higher LD with the markers than the true QTL would be, with the same consequence of favoring GS over PS, but not obviously one GS scheme over another. The present simulations were conducted without regard to the fact that the base population for any real GS will have been under phenotypic selection for some time. By virtue of the Bulmer effect, such selection will generate repulsion-phase linkage disequilibria between QTL, reducing the genetic variance and increasing the difficulty of QTL detection. Furthermore, no mutation model was applied to the simulations, and results relate strictly to standing variation at the start of selection. Phenotypic selection benefits from mutational variation (reviewed in [22]), but it is not clear how GS might, considering that new mutant effects will not immediately be present in the training population. Finally, on a simple note, the breeding schemes used here assumed that GS reduced breeding cycle times only by half. In practice, for crops [12,23] and livestock [24] the reduction is likely to be much greater than that, favoring GS over PS more than predicted here.

Given so many caveats, the value of these simulations is clearly not to accurately predict relative responses of different breeding schemes over long-term selection but to ask whether GS can work over the long-term, to raise hypotheses relative to its success or failure, and to point to possible solutions to be tested empirically. In those regards, the stochastic simulations provide three primary observations and a number of insights into the mechanisms causing them. The observations are: 1) by selecting prior to phenotyping, GS allows a more rapid initial gain than is possible under PS or GPS; 2) while these gains are occurring, UGS is also rapidly losing favorable QTL alleles such that UGS reaches a selection plateau early on; 3) long-term gain can be increased, with little sacrifice on short-term gain, by selecting on a criterion that weights more heavily favorable marker alleles at low frequency. There is nothing surprising about observation 1. This result has been anticipated since the invention of GS [1] and has been the cause of much excitement since GS became practically feasible [9,24]. The second observation is more problematic and had not been anticipated by deterministic simulations of GS [25]. Habier et al. [10] have shown that GS captures not just marker - QTL associations but also genetic relationships via marker information [see also [5] and [11]]. Thus, GS is prone to the selection of close relatives that occurs in standard animal-model BLUP [26]. The theory has predicted that GS should reduce rates of inbreeding compared to selection on breeding value BLUP [27]. This claim is not disputed here, since no simulation of BLUP selection was performed. The theory is based on the extent to which the selection criterion is able to predict the Mendelian sampling term (i.e., within-family effects). In the absence of phenotyping prior to selection, animal model BLUP estimation provides no prediction of the term whereas GS does. In fact, as the GS model becomes more accurate, it can better predict the term, its reliance on genetic relationship information decreases, and inbreeding under GS decreases. Confirmation of that dynamic is apparent in the opposing trends of Figures 3B and 3D: when the model has just been updated with newly-measured phenotypes, it is more accurate (Figure 3D) and the rate of inbreeding is decreased (Figure 3B); conversely, during selection in off-seasons without model updating, the rate of inbreeding is increased. Likewise, but over a period of many seasons, as the accuracy of GS gradually decreases, the rate of inbreeding under GS gradually increases. The opposite effect would be expected under phenotypic selection: as genetic variance is depleted and heritability declines, PS accuracy would decline and selection would become random. In that case, the rate of inbreeding should converge toward 0.05 per generation, as would be expected under random-mating with 20 gametes (or completely inbred diploids) selected in each generation.

There is, nevertheless, disagreement between the present finding of increasing rate of inbreeding under GS with decreasing GS accuracy and the prediction from selection index theory that rate of inbreeding should be insensitive to accuracy [25]. Presumably, this disagreement has to do with the use of genetic relationship information by GS that is not accounted for by the theory. But the meaning of "use of genetic relationship information" is not particularly clear. This mechanism may occur: allele effect estimates used in GS are influenced by the regression of family means on within-family allele frequency. These estimates would contribute to accuracy by improving predictions of family means, but would contribute nothing to the estimation of Mendelian sampling terms. Thus they increase between-family but not within-family variance of predictions. Finally, as the overall accuracy of GS decreases, the importance of this family-mean prediction component increases, and with it the correlation between GS predictions for relatives. When applying index selection theory to GS, however, the analysis assumes that the variance of the GS prediction is split equally between within- and between-family effects, regardless of accuracy.

The fact that a very simple weighting scheme can greatly increase long-term gain with little loss in short-term gain is probably the most exciting observation made here. Goddard [18] have proposed and Hayes et al. [6] have clarified differentially weighting markers to increase weight on favorable low-frequency alleles. All other things equal, UGS should be more accurate than WGS. This higher accuracy can be seen in the very first selection cycle, because initial conditions are the same for all methods (Figure 3D). Rapidly thereafter, however, WGS catches up because strong selection on low frequency favorable alleles boosts genetic variance (Figure 3A), leading to proportional increases in gain. This observation causes concern as to the generality of the benefit of the weighting scheme across different genetic models. In the model used here, each QTL generated equal variance so allele substitution effects were inversely related to the square root of the variance of QTL allelic states. In other words, QTL with low minor allele frequencies had large allele substitution effects. This genetic model may not be unrealistic for a population under stabilizing selection [28]. For a population under directional selection, deleterious alleles with large substitution effects would be expected to be at low frequencies. In addition, breeders should be most concerned with capturing new favorable mutations when they are at a low frequency [22]. But clearly, this genetic model is also ideal for the weighting scheme outlined here: low frequency marker alleles that are heavily weighted will more often be associated with large substitution QTL that will generate large gain. To test the impact of the genetic model, the simulations shown in Figure 2 were also run using a genetic model where the QTL allele substitution effect was sampled at random (ignoring QTL allele frequency) from a standard normal distribution. Under the random model, the weighting scheme was still beneficial over the long term, increasing final gain by 10% to 15% (14% average) over UGS, depending on heritability, TP size, and phenotyping scheme. In comparison, under the original equal-variances model, the range of improvement was 14% to 28% (22% average). In other respects, the progression of genetic gain was remarkably similar across genetic models (Additional file 1, Figure S1). Thus, the advantage of WGS observed does not depend on an inverse relationship between QTL allele frequency and effect size, though its robustness to other aspects of the genetic model is still subject to research. Finally, to further diminish the small loss of initial gain under WGS relative to UGS, it would be possible to choose one set of lines for potential variety release using UGS while selecting a different set to become parents of the next generation of progeny candidates using WGS [9,24]. In some sense, UGS reflects the current genetic value of a line while WGS reflects its potential for long-term contribution to the breeding program.

The mechanism of WGS is manifest in three other ways. First, the rate of inbreeding on the basis of pedigree was lower for WGS than UGS (Figure 3B). This lower rate of inbreeding was not caused by a greater accuracy of WGS than UGS: for about the first half of the seasons simulated, WGS had a lower accuracy than UGS. It is difficult to see why weighting low-frequency favorable alleles would differentially affect the between-family versus within-family variances of the predictor. Rather, the higher genetic variance present under WGS than under UGS would simply lead to more accurate allele effect estimates generally, which would in turn affect those variances. Second, WGS fixes markers more slowly than UGS (Figure 3G). Consequently, markers close to QTL remain polymorphic for much longer in WGS than in UGS (Figure 3F), and WGS retains markers in higher LD with the QTL than does UGS (Figure 3E). This causal sequence presumably also plays a role in lifting the accuracy of WGS above that of UGS in the second half of the seasons simulated (Figure 3D). Naturally, the greater genetic variance generated and preserved by WGS than UGS would increase the heritability of observations in the TP, also improving model accuracy. Third, and perhaps most importantly, WGS loses fewer favorable alleles than UGS (Figure 3H). The rare marker alleles that WGS weights more heavily are in higher LD with rare QTL alleles than other markers. The risk of losing the QTL alleles is therefore indirectly reduced by this weighting. Note that these reasonings concerning WGS assume a simple situation with one marker in LD with one QTL. In reality, the effect of a QTL may be absorbed by several markers in partial LD with it. Nevertheless, those markers are likely to have similar allele frequencies as the QTL such that the essential mechanism remains valid.

## Conclusions

What occurs initially upon adoption of GS should matter most to current plant and animal breeders, because that is what is happening in breeding programs now. Even assuming optimistic breeding cycle times, the long-term predictions presented here are about 20 years away, at which point breeding technologies will no doubt have changed dramatically. But even in the first cycles, the benefits of a large TP and of WGS are evident in the form of the reduction of favorable alleles lost from the breeding population (Figure 3H). Some of these alleles will inevitably be lost because they are in low LD with any marker. Indeed, Figure 3E shows a slight increase in the mean QTL-marker LD after the first generation of selection. That increase is due to the fact that some low-frequency, low LD alleles are lost immediately and they therefore no longer enter the mean. Retaining those alleles would be difficult and would likely cause unwarranted losses of selection gain. Nevertheless, it appears that WGS goes some way in the right direction, and further research on its optimization is warranted. In general, loss of genetic diversity will rise in tandem with the greater number of selection cycles made possible by GS, suggesting that methods that balance selection gain with the maintenance of diversity [29] should be a priority.

## Competing interests

The author declares that they have no competing interests.

## Supplementary Material

Additional file 1 **Figure S1**. Identical to Figure 2, save that the genetic model included QTL effects sampled from a standard normal distribution Click here for file
