# Supplementary figures and images for: Dynamics of long-term genomic selection
Source: Genet Sel Evol. 2010 Aug 16;42(1):35. doi: 10.1186/1297-9686-42-35 (PMC2936280; doi:10.1186/1297-9686-42-35)

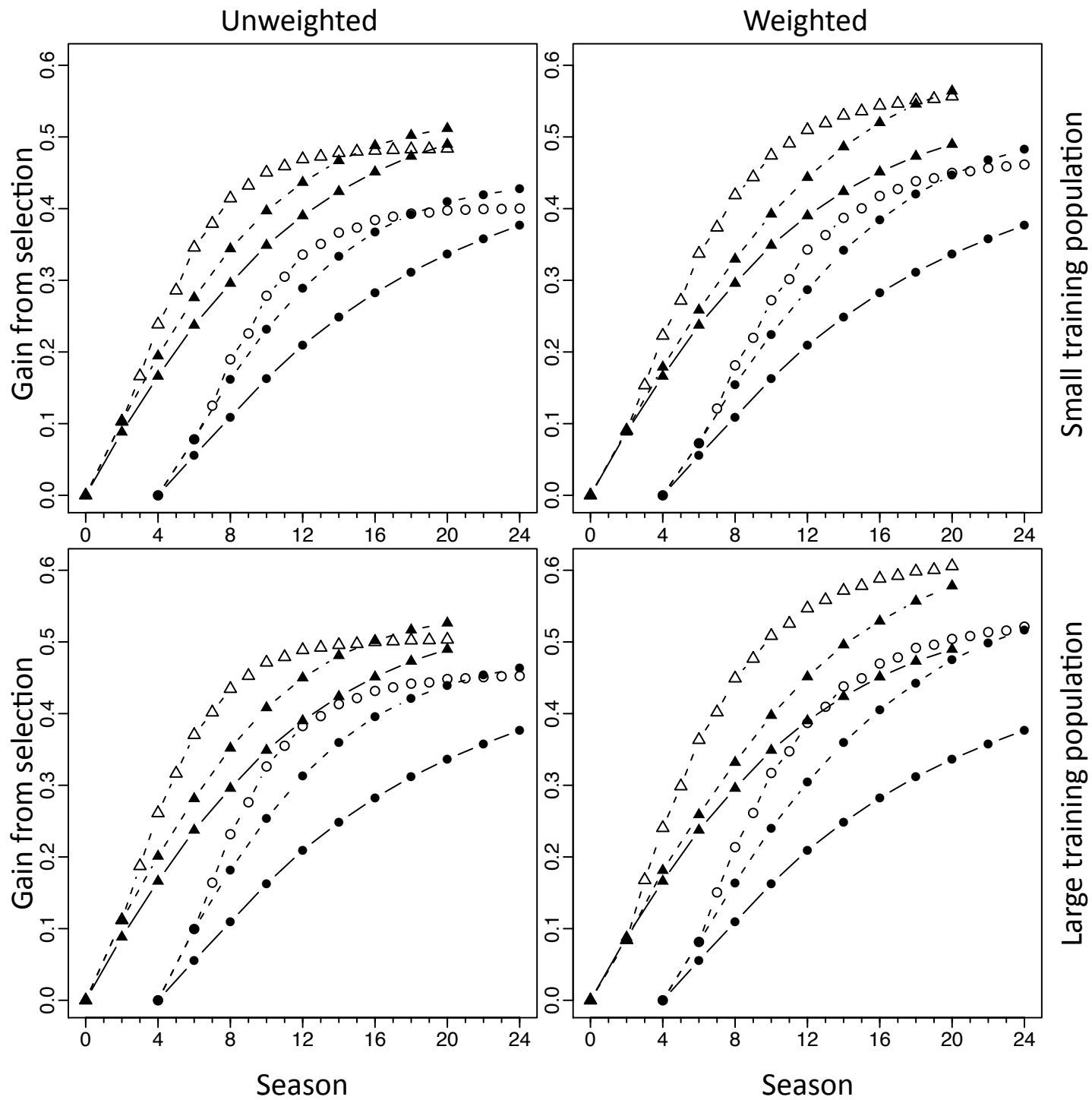

Supplement: Additional file 1 — Figure S1. Identical to Figure 2, save that the genetic model included QTL effects sampled from a standard normal distribution [file 1297-9686-42-35-S1.PDF]
